# A probe into the acid deposition mitigation path in China over the last four decades and beyond

**DOI:** 10.1093/nsr/nwae007

**Published:** 2024-01-05

**Authors:** Qian Yu, Xiaodong Ge, Haotian Zheng, Jia Xing, Lei Duan, Dongwei Lv, Dian Ding, Zhaoxin Dong, Yisheng Sun, Posch Maximilian, Danni Xie, Yu Zhao, Bin Zhao, Shuxiao Wang, Jan Mulder, Thorjørn Larssen, Jiming Hao

**Affiliations:** State Key Laboratory of Environmental Simulation and Pollution Control, School of Environment, Tsinghua University, Beijing 100084, China; State Key Laboratory of Pollution Control & Resource Reuse and School of the Environment, Nanjing University, Nanjing 210023, China; State Key Laboratory of Environmental Simulation and Pollution Control, School of Environment, Tsinghua University, Beijing 100084, China; State Key Laboratory of Environmental Simulation and Pollution Control, School of Environment, Tsinghua University, Beijing 100084, China; State Key Laboratory of Environmental Simulation and Pollution Control, School of Environment, Tsinghua University, Beijing 100084, China; State Environmental Protection Key Laboratory of Sources and Control of Air Pollution Complex, School of Environment, Tsinghua University, Beijing 100084, China; State Key Laboratory of Environmental Simulation and Pollution Control, School of Environment, Tsinghua University, Beijing 100084, China; State Environmental Protection Key Laboratory of Sources and Control of Air Pollution Complex, School of Environment, Tsinghua University, Beijing 100084, China; State Key Laboratory of Environmental Simulation and Pollution Control, School of Environment, Tsinghua University, Beijing 100084, China; State Key Laboratory of Environmental Simulation and Pollution Control, School of Environment, Tsinghua University, Beijing 100084, China; State Key Laboratory of Environmental Simulation and Pollution Control, School of Environment, Tsinghua University, Beijing 100084, China; State Key Laboratory of Environmental Simulation and Pollution Control, School of Environment, Tsinghua University, Beijing 100084, China; State Environmental Protection Key Laboratory of Sources and Control of Air Pollution Complex, School of Environment, Tsinghua University, Beijing 100084, China; International Institute for Applied System Analysis (IIASA), Laxenburg A-2361, Austria; School of Land Engineering, Chang'an University, Xi'an 710064, China; State Key Laboratory of Environmental Simulation and Pollution Control, School of Environment, Tsinghua University, Beijing 100084, China; State Key Laboratory of Pollution Control & Resource Reuse and School of the Environment, Nanjing University, Nanjing 210023, China; State Key Laboratory of Environmental Simulation and Pollution Control, School of Environment, Tsinghua University, Beijing 100084, China; State Key Laboratory of Pollution Control & Resource Reuse and School of the Environment, Nanjing University, Nanjing 210023, China; Faculty of Environmental Sciences and Natural Resource Management, Norwegian University of Life Sciences, Ås 5003, Norway; Norwegian Institute for Water Research, Oslo 0349, Norway; State Key Laboratory of Environmental Simulation and Pollution Control, School of Environment, Tsinghua University, Beijing 100084, China; State Key Laboratory of Pollution Control & Resource Reuse and School of the Environment, Nanjing University, Nanjing 210023, China

**Keywords:** acid deposition, critical loads, acidification, eutrophication, ammonia abatement

## Abstract

China currently has the highest acid deposition globally, yet research on its status, impacts, causes and controls is lacking. Here, we compiled data and calculated critical loads regarding acid deposition. The results showed that the abatement measures in China have achieved a sharp decline in the emissions of acidifying pollutants and a continuous recovery of precipitation pH, despite the drastic growth in the economy and energy consumption. However, the risk of ecological acidification and eutrophication showed no significant decrease. With similar emission reductions, the decline in areas at risk of acidification in China (7.0%) lags behind those in Europe (20%) or the USA (15%). This was because, unlike Europe and the USA, China's abatement strategies primarily target air quality improvement rather than mitigating ecological impacts. Given that the area with the risk of eutrophication induced by nitrogen deposition remained at 13% of the country even under the scenario of achieving the dual targets of air quality and carbon dioxide mitigation in 2035, we explored an enhanced ammonia abatement pathway. With a further 27% reduction in ammonia by 2035, China could largely eliminate the impacts of acid deposition. This research serves as a valuable reference for China's future acid deposition control and for other nations facing similar challenges.

## Teaser

This paper presents the status, controls, and impacts of acid deposition in China, and explores a path for acid deposition mitigation with the simultaneous goals of air quality improvement and carbon reduction in the future.

## INTRODUCTION

**Figure 1.**
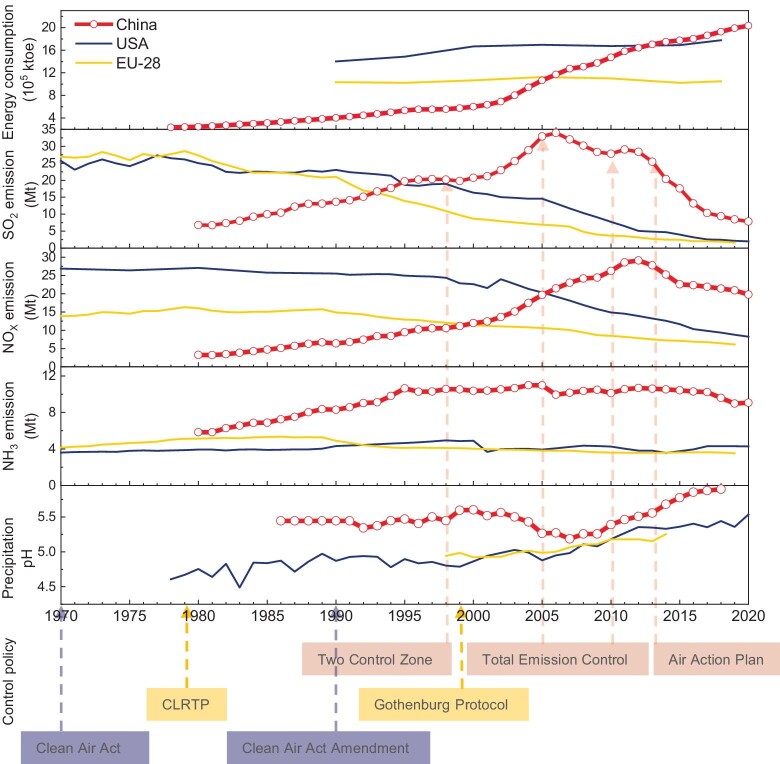
Trends of acid rain development and related variables in China compared with Europe (EU-28) and the USA.

Acid deposition, encompassing sulfur (S) and nitrogen (N) deposition, emerged as a most pressing environmental concern in Europe and North America during the late twentieth century [1–4]. This issue led to the widespread acidification and eutrophication of terrestrial and aquatic ecosystems, causing damage to biological diversity and health [1,3,5,6]. With the slowdown in economic growth and the implementation of policy actions for decades, e.g. the 40-year Convention on Long-range Transboundary Air Pollution (CLRTAP) in Europe and the 40-year Clean Air Act Amendments in the USA, emissions of the key acidifying pollutants, namely sulfur dioxide (SO_2_) and nitrogen oxides (NO_X_), have been reduced by >90% and >60% since 1980, respectively (Fig. 1). This success has spurred renewed research interest in assessing the current status of acid deposition and its environmental impacts, aiming to evaluate the efficacy of these policies and inform future legislative initiatives [7].

One of the foremost scientific frameworks for evaluating ecological impacts associated with atmospheric emissions and subsequent deposition is the concept of critical loads (CLs), reflecting the strong interactions of science and policy. The CL is defined as a quantitative estimate of exposure to deposition, below which significant harmful effects on sensitive elements of the environment do not occur according to current knowledge [8]. The risk is thereby expressed through CL exceedance, i.e. representing the extent to which deposition surpasses the established critical load. The CL approach has been applied in negotiating several protocols for the CLRTAP (e.g. the Second Sulphur Protocol and the Gothenburg Protocol) to develop advanced strategies for emission abatement [8]. Analogous to public health standards, the CL is considered a long-term objective for emission control policies from the view of ecological health.

Driven by rapid economic growth and increased fossil fuel combustion, China's emissions of SO_2_ and NO_X_ surged by over three and seven times, respectively, within a 30-year span from 1980 [9–11] (Fig. 1, and Fig. S1 in the Supplementary Data online). Consequently, the peak emissions of SO_2_ (∼33 Mt yr^−1^ in 2006) and NO_X_ (∼29 Mt yr^−1^ in 2012) in China surpassed those in Europe and the USA, leading to heavier acid deposition [9–11] (Fig. 1). Similarly, China has made great efforts to control air pollutant emissions. Gradually stricter policies for emission abatement of the acidifying precursors have been launched in China over the past 20 years, including the designation of the SO_2_ Pollution Control Zone and Acid Rain Control Zone (Two Control Zones) in 1998, the Total Emission Control of SO_2_ and NO_X_ (setting compulsory targets to achieve the national goal of 10% reduction in the SO_2_ emission from 2005 to 2010, and further reduce SO_2_ and NO_X_ emissions by 8% and 10% by 2015, respectively), the Action Plan of Air Pollution Prevention and Control (Air Action Plan, setting 15% reduction of national SO_2_ and NO_X_ emissions by 2020) and the Three-Year Action Plan to Fight Air Pollution (Fig. 1 and Fig. S1). By far, however, China lacks efficiency assessments for control strategies acting on acid deposition and its ecological impacts [12–14]. To elucidate the status, causes and controls of acid deposition, we compiled data spanning 1980–2020 on China's national economy, energy consumption, emissions, deposition, precipitation pH and control policies. Furthermore, we evaluated the efficiency of these control strategies on ecological health improvement using the CL concept, both retrospectively and in future scenarios. Through a comprehensive comparative analysis with Europe and the USA, we explored a pathway to mitigate the ecological impacts of acid deposition.

## RESULTS AND DISCUSSION

### Acid-rain-mitigation benefit of emission abatements

It should be affirmed that the abatement measures had success in acid rain mitigation in China. By 2020, national emissions of SO_2_ and NO_X_ had seen substantial reductions of 77% and 32%, respectively, compared with their peak values in 2006 and 2012 (Fig. 1). Consequently, the national average pH of precipitation has continually increased since 2007 and exceeded 5.6 after 2014. Notably, the proportion of acid rain areas (i.e. the area with precipitation pH < 5.6) dwindled from >40% around the peak of acid deposition in 2005 to ∼12% in 2020 (data from China Meteorological Administration 1992–2020, http://s.cma.gov.cn/zfxxgk/gknr/qxbg/). Furthermore, only 4.8% of the country experienced acid rain in China in 2020 according to the Bulletin on the State of China's Ecological Environment in 2020 (https://www.mee.gov.cn/hjzl/sthjzk/zghjzkgb/). Although different networks may have varying reports on the proportion of acid rain areas due to different distributions of monitoring sites, overall, there has been a significant decrease in acid rain areas in China. Compared with Europe and the USA, China has achieved a more rapid decline in SO_2_ emissions and a sharper increase in precipitation pH since 2005 (Fig. 1). Remarkably, the trajectories of acidifying pollutant emissions and social-economic development have become progressively decoupled. Despite significant growth in the gross domestic product (GDP) (by 5.4 times), energy consumption (by 64%) and vehicle volume (by 7.7 times) since 2005, when SO_2_ began to decline (Fig. S1), the trends in emissions have notably diverged.

Compared with Europe and the USA, China have had much larger emissions of SO_2_ and NO_2_ since 2005 (Fig. 1). However, China always has a much higher annual average precipitation pH and a lower incidence of acid rain areas (4.8% in China vs. 67% in the USA, Table S1). This divergence is likely attributed to the robust acid-neutralizing capacity provided by larger anthropogenic emissions of base cations (Bc, i.e. Ca^2+^, Mg^2+^ and K^+^; ∼2.1 times that of the USA, Fig. S1) and ammonia (NH_3_, ∼2.6 times that of the USA, Fig. 1) [10,11,15,16]. With the control of fine particle matter (PM_2.5_) in China, the deposition of Bc has declined [17], but less than the reductions in SO_2_ and NO_X_. Concurrently, NH_3_ emissions have remained stable without control policies. The dissolution of NH_3_ in rainwater forms ammonium (NH_4_^+^) and buffers acidity, thus increasing the precipitation pH. However, NH_4_^+^ is another important acidifying compound due to the intense proton (H^+^) release during assimilation and nitrification once NH_4_^+^ deposits into ecosystems [16]. Thus, the increase in precipitation pH does not adequately reflect the mitigation of acid deposition and its ecological impacts. The current national average S and N depositions in China are still much larger than the peak values in Europe and America and have shown a limited decline in recent years (Fig. S1) [18–21]. This result implies the continuing potential risk of acidification and eutrophication of ecosystems. In addition to precipitation pH, more evidence should be provided to show that control measures lead to the intended improvements in terrestrial and freshwater ecosystems.

### Ecological impacts alleviation from past to future

**Figure 2.**
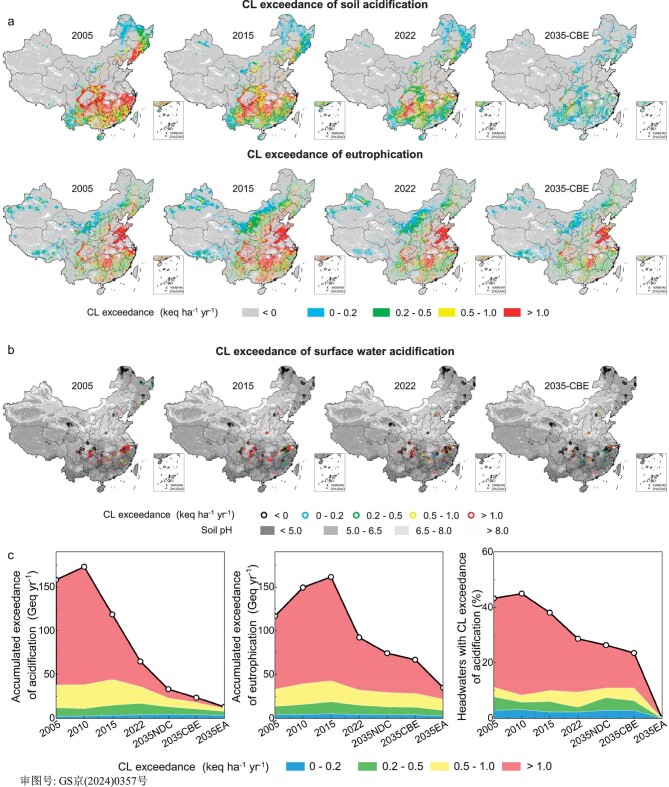
Critical loads (CLs) exceedance with the changes in acid deposition. (a) Distributions of CL exceedances in 2005, 2015, 2022 and 2035-CBE scenarios for soil acidification and ecosystem eutrophication. (b) Distributions of CL exceedances for surface water acidification. (c) Accumulated amounts of CL exceedances.

We updated CLs for soil acidification and eutrophication (Fig. S2) as well as CLs for surface water acidification (Fig. S3), and subsequently computed the historical CL exceedances with the simulations of acid deposition in 2005, 2010, 2015 and 2022 (Fig. 2). Specifically, we selected the depositions in 2005 and 2010, corresponding to the commencement of the national Total Emission Control of SO_2_ and NO_X_, as historical depositions, respectively. The depositions of 2015 and 2022 was considered representative of the current deposition. Overall, environmental risk assessments based on CL exceedances indicated that the emission reductions from 2005 to 2015 caused a limited or non-significant decline in the degree and extent of ecosystem acidification (Fig. 2). For instance, although the accumulated exceedance for soil acidification decreased from 158 Geq yr^−1^ in 2005 to 119 Geq yr^−1^ in 2015, the total area with CL exceedance for soil acidification showed no significant changes from 2005 to 2015 (accounting for 15.9% and 16.1% of the country in 2005 and 2015, respectively). Additionally, there were no significant changes in the percentage of streams that CLs exceeded, and nearly 40% of the headwaters in China faced the risk of acidification during the 2005–15 periods. Meanwhile, instead of decreasing, the areas in which the CL of nutrient N was exceeded and accumulated exceedance for eutrophication increased gradually from 2005 to 2015, illustrating the increased risk of ecological eutrophication in China. In 2015, the total area with CL exceedance for eutrophication accounted for >20% of the country. In contrast, from 2015 to 2022, there was significant alleviation in the ecological impacts of acid deposition, particularly in soil acidification and eutrophication. Nevertheless, the total area with CL exceedances still accounted for ∼14% and 15% of the country for soil acidification and eutrophication, respectively, under the deposition in 2022. This underscores the current high risk of ecological impacts from acid deposition in China.

At present, the Chinese government is devoting great efforts to simultaneously improving air quality and mitigating climate change, adopting the ‘Beautiful China’ and ‘Carbon Peak and Neutral’ strategies in 2020. These strategies aim to achieve ambient PM_2.5_ concentrations of ≤35 μg m^−3^ for all cities by 2035 and attain carbon dioxide (CO_2_) emission peaks before 2030, respectively. Pathways to meeting these dual targets have been extensively explored [22–24], including the 2035-NDC scenario, aligning with nationally determined contributions outlined in the Paris Agreement, and the 2035-CBE scenario, incorporating co-benefit energy policies and end-of-pipe controls with maximum feasible reductions in 2035 [24]. Under the 2035-NDC scenario, national emissions of SO_2_, NO_X_ and NH_3_ are projected to decrease by 62%, 51% and 18%, respectively, compared with those in 2015. The 2035-CBE scenario envisions even more substantial reductions, with emissions of SO_2_, NO_X_ and NH_3_ dropping by 78%, 70% and 18%, respectively (Fig. S4) [24]. Consequently, S deposition is expected to decline by >50% in most regions, except in areas with low deposition, such as northwest China and the Tibet Plateau (Fig. S4). Although the decrease in N deposition is less pronounced due to the challenges in controlling ammonia emissions, there is a sharp national decline, particularly in the north and parts of northwest China (Fig. S4). Under the acid deposition in the 2035-CBE scenario, the area and amount of CL exceedance for soil acidification decreased significantly to only 8.9% of the country and 23.4 Geq yr^−1^, respectively (Fig. 2). Concurrently, compared with 2015, the percentage of surface water with CL exceedance for acidification decreased by 16% and the proportion of area with CL exceedance for eutrophication decreased by 7.4%. Nevertheless, nearly a quarter of the headwaters and 13% of the area in China will remain at risk of acidification and eutrophication, even under the 2035-CBE scenario. This result shows that China cannot mostly eliminate (i.e. the area with CL exceedances being <10% of the country) the ecological impacts of acid deposition in the future, even with the achievement of the dual targets of both air quality and CO_2_ mitigation.

**Figure 3.**
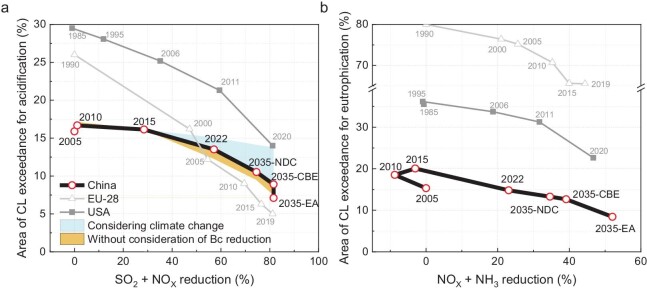
Changes in the percentage of area with critical loads (CLs) exceedance with emission reductions. (a) For soil acidification. The shadow above the line shows the CL exceedance increase considering climate change in the future and the shadow below the line shows the changes in CL exceedance without considering the changes in Bc deposition. (b) For eutrophication. In China, the emission reductions were compared to those in 2005, while for the EU-28 and the USA, they were compared to those in 1990 and 1985, respectively.

It is essential to highlight that the anticipated reduction in Bc deposition with PM_2.5_ pollution control would cause a decrease in the computed CLs for acidification (Fig. S5). Consequently, the mitigation of CL exceedances for acidification may proceed at a slower pace, taking into account the projected reduction in Bc deposition in the future (Fig. 3a). In contrast, the CL of N is less affected, primarily due to its significant dependence on nutrient N rather than acidifying N (Fig. S5). Furthermore, future climate change poses an additional challenge to the mitigation of ecological impacts from acid deposition. According to the prediction of future climate change [25], most regions in China are expected to experience increases in soil temperature and moisture. Although the rise in temperature marginally accelerates weathering rates (<10%), the concurrent increase in plant uptake is projected to significantly elevate Bc uptake (up to 40%) (Fig. S6). Consequently, substantial parts of China demonstrate decreasing CLs of acidification when considering climate change. Calculations indicate that the proportion of area with the CL exceedance for acidification will be 55% larger than that without considering future climate change (Fig. 3a).

### Differences between China and Europe or the USA

Under peak acid deposition, the proportion of the area at risk of soil acidification in China in 2005 (∼16%) was significantly smaller than that in Europe (∼26%) and the USA (∼29%) in approximately 1990 (Fig. 3a). This result indicates that the degree of ecosystem acidification is relatively lower in China, even under relatively higher acid deposition (Fig. S1). Moreover, the CL exceedance of nutrient N in China has always been much lower than that in Europe and the USA (Fig. 3b), indicating that China faces a lower risk of ecological eutrophication. The lower risks of ecological acidification and eutrophication are attributed to the much higher CLs in China resulting from higher Bc deposition, vegetation uptake, sulfate retention and denitrification ratios (see Supplementary Data online). Nevertheless, the emission reductions had relatively poorer efficiency in reducing the CL exceedances for acidification and eutrophication in China. Under the 2035-CBE scenario, China's emission reductions of SO_2_ and NO_2_ since 2005 will be similar to those in Europe and the USA from 1990 to 2020 (Fig. 3a). However, the decline in the area with CL exceedances for acidification in China (decreased by ∼7.0%) was far less than that in Europe (decreased by ∼20%) and the USA (decreased by ∼15%) (Fig. 3a). Similarly, with a similar reduction in the sum of NO_2_ and NH_3_ emissions, the proportion of area with CL exceedances for eutrophication showed a lower decrease in China from 2010 to the 2035-CBE scenario (∼7.4%) compared with Europe (14%) and the USA (13%).

Reducing the area and amount of CL exceedances guided the government policy on reducing emissions of acidifying pollutants in Europe and North America [7]. In contrast, the emissions reduction was aimed at the improvement of air quality in China, resulting in the region with the largest emission reductions being located in the more industrial eastern China, which has relatively high CLs due to the already destroyed natural vegetation (Fig. S2). However, the reduction in atmospheric deposition was not sufficient in southern China with high CL exceedances for acidification and in the northwestern region with a large area of CL exceedance for nutrient N (Fig. 2a and Fig. S6c). Regarding the spatial distribution, the CL exceedance for acidification decreased in some parts of eastern China but increased in some parts of western China from 2005 to 2015 (Fig. 2a). This is mainly because S deposition declined in most parts of eastern China but increased in some parts of western China (Fig. S4). Therefore, abatement strategies with the goal of improving air quality in China have relatively lower efficiency in the mitigation of environmental impacts induced by acid deposition. The CL-based policies for emission abatement in Europe have been shown to be most cost-effective in the three regions where emissions reductions have achieved significant success. This result suggests that reducing CL exceedances (i.e. ecological sensibility) should be considered one of the main objectives for further emissions reduction in China.

### Pathway for acid deposition mitigation in the future

Given the large areas at risk of eutrophication (>13% of the country) under the 2035-CBE scenario, N pollutants were regarded as the primary control objective during the exploration of the pathways for long-term targets of further acid deposition alleviation in China. Under the 2035-CBE scenario, the emissions of NH_3_ will only decrease by 18% compared with 2015 because the NH_3_ emissions are very hard to control by currently available end-of-pipe control technologies [24]. However, according to the prediction of NH_3_ emissions in China, there is still great potential for NH_3_ reduction (reduce by 30%–50%) after the implementation of both end-of-pipe control technologies during industrial production and feasible abatement measures during agricultural activities [16]. Here, an enhanced ammonia abatement pathway (2035-EA scenario) was explored to drastically reduce acid deposition and its ecological impacts. Specifically, we set a 40% reduction in NH_3_ emissions from 2015 and the key control zone was identified by the CL exceedance for nutrient N (2035-EA scenario). It means a further reduction of NH_3_ emissions by ∼27% compared with the 2035-CBE scenario (Fig. S7). The regions and degrees of potential risk for soil acidification and eutrophication were identified based on the distribution of their CL exceedance under the 2035-CBE scenario.

Under the 2035-EA scenario, the area where N deposition exceeds the CL of nutrient N will sharply decrease to only 8.0% of the country (Fig. 3b). Additionally, the area with acidity CL exceedances for soil will decrease to only 7.1% of the country (Fig. 3a) and the percentage of headwaters with CL exceedances for acidification will decrease to near zero (Fig. 2c). This means that >92% of the country could be protected from acidification and eutrophication, and almost all the headwaters could avoid acidification, with a further 27% reduction in NH_3_. Meanwhile, the enhanced NH_3_ reduction would contribute to the further mitigation of PM pollution. Under the 2035-EA scenario, the average PM_2.5_ concentrations will decrease by an extra 12%.

Our findings emphasize that the sensitivity of the ecosystem to acid deposition should be considered when formulating effective regional policies to achieve multiple long-term targets of air quality improvement, CO_2_ mitigation and acid deposition alleviation. This insight could serve as a valuable reference for other regions, such as India, Southeast Asia, South America and South Africa, which currently experience or will face chronically elevated acid deposition and thus acidification and eutrophication as a consequence of their industrial and economic development [26,27]. Notably, these regions share similarities with China, being located in tropical or subtropical regions, and may experience relatively less severe eutrophication compared with Europe and North America. Consequently, the explored pathway in this study for China, focusing on NH_3_ emission reductions guided by CL exceedances, can offer a particularly important reference for these regions.

## MATERIALS AND METHODS

### Data compilation

The emissions of SO_2_, NO_X_ and NH_3_ from 1980 to 2020 were the result of the Multi-resolution Emission Inventory for China (http://www.meicmodel.org) [28,29]. Trends of S and N deposition were summarized from the published data and measurements across China (see Method in the Supplementary Data for details). The Bc deposition is the simulation value using a multi-layer Eulerian model (only Ca^2+^ and Mg^2+^) [17]. Additionally, the trends of precipitation pH and its distributions were derived from 74 monitoring sites across China of the China Meteorological Administration (CMA; 1992–2020; http://s.cma.gov.cn/zfxxgk/gknr/qxbg/). Data on the GDP, energy consumption, coal consumption, vehicle volume and fertilizers application were derived from the National Bureau of Statistics of China (https://www.stats.gov.cn/). For details of the data for emissions, depositions and CLs in Europe and the USA, see Method in the Supplementary Data.

### CLs

We used the Simple Mass Balance model to update the national CLs for soil acidification and eutrophication with 1 × 1 km^2^ resolution. The maximum CL of sulfur (CL_max_(S)) is derived as in [30]:


(1) \begin{eqnarray*}
{\mathrm{C}}{{\mathrm{L}}}_{{\mathrm{max}}}{\mathrm{(S)\ = \ B}}{{\mathrm{c}}}_{{\mathrm{dep}}} + {\mathrm{B}}{{\mathrm{c}}}_{\mathrm{w}} - {\mathrm{B}}{{\mathrm{c}}}_{\mathrm{u}} - {\mathrm{AN}}{{\mathrm{C}}}_{{\mathrm{le,\mathrm{crit}}}}
\end{eqnarray*}

where the subscript dep stands for deposition, and subscripts w and u mean soil weathering and net uptake by vegetation, respectively. Bc stands for base cations except for Na. ${\mathrm{AN}}{{\mathrm{C}}}_{{\mathrm{le, crit}}}$ is the critical leaching of acid-neutralizing capacity, which can be calculated using the following equation:


(2) \begin{eqnarray*}
{\mathrm{AN}}{{\mathrm{C}}}_{{\mathrm{le,\mathrm{crit}}}} &&= - \frac{{{\mathrm{1}}{\mathrm{.5}}\left( {{\mathrm{B}}{{\mathrm{c}}}_{{\mathrm{dep}}}{\mathrm{ + \ B}}{{\mathrm{c}}}_{\mathrm{w}}\ - {\mathrm{\ B}}{{\mathrm{c}}}_{\mathrm{u}}} \right)}}{{{{\left( {\frac{{{\mathrm{Bc}}}}{{{\mathrm{Al}}}}} \right)}}_{{\mathrm{crit}}}}}\\
&& -\, {Q}^{\frac{{\mathrm{2}}}{{\mathrm{3}}}}{\mathrm{\ \times \ }}{\left[ {\frac{{{\mathrm{1}}{\mathrm{.5}}\left( {{\mathrm{B}}{{\mathrm{c}}}_{{\mathrm{dep}}}{\mathrm{ + \ B}}{{\mathrm{c}}}_{\mathrm{w}}\ - {\mathrm{\ B}}{{\mathrm{c}}}_{\mathrm{u}}} \right)}}{{{{\left( {\frac{{{\mathrm{Bc}}}}{{{\mathrm{Al}}}}} \right)}}_{{\mathrm{crit}}}{\mathrm{\ \times \ }}{K}_{{\mathrm{gibb}}}}}} \right]}^{\frac{{\mathrm{1}}}{{\mathrm{3}}}} \!\! \\
\end{eqnarray*}

where *Q* is the runoff flux leaving the root zone and ${K}_{{\mathrm{gibb}}}$ is the Gibbsite constant describing the balance between Al^3+^ and H^+^.

The minimum CL for nitrogen is derived as:


(3) \begin{eqnarray*}
{\mathrm{C}}{{\mathrm{L}}}_{{\mathrm{min}}}{\mathrm{(N) =\, }}{{\mathrm{N}}}_{\mathrm{u}}{\mathrm{\ + \ }}{{\mathrm{N}}}_{\mathrm{i}}
\end{eqnarray*}

where *N*_u_ and *N*_i_ stand for the net nitrogen uptake and immobilization, respectively. The maximum CL for nitrogen is derived as:


(4) \begin{eqnarray*}
{\mathrm{C}}{{\mathrm{L}}}_{{\mathrm{max}}}{\mathrm{(N)\ = \ C}}{{\mathrm{L}}}_{{\mathrm{min}}}{\mathrm{(N)\ + \ C}}{{\mathrm{L}}}_{{\mathrm{max}}}{\mathrm{(S)/(1}} - {f}_{{\mathrm{de}}}{\mathrm{)}}
\end{eqnarray*}

where *f*_de_ is the fraction of nitrogen net input (= N_dep_ − CL_min_(N)) that is denitrified.

In addition to acidification, excess nitrogen can also lead to eutrophication. The CL of nutrient nitrogen (or CL of eutrophication) is derived as:


(5) \begin{eqnarray*}
{\mathrm{C}}{{\mathrm{L}}}_{{\mathrm{eut}}}{\mathrm{(N)}} = {{\mathrm{N}}}_{\mathrm{u}}{\mathrm{\ + \ }}{{\mathrm{N}}}_{{\mathrm{i\ }}}{\mathrm{ + \ }}{{\mathrm{N}}}_{{\mathrm{le,\mathrm{crit}}}}{\mathrm{/(1}} - {f}_{{\mathrm{de}}}{\mathrm{)}}
\end{eqnarray*}

where N_le, crit_ is the accepted nitrogen leaching. The CL of nitrogen is defined as the minimum of ${\mathrm{C}}{{\mathrm{L}}}_{{\mathrm{max}}}{\mathrm{(N)}}$ and ${\mathrm{C}}{{\mathrm{L}}}_{{\mathrm{eut}}}{\mathrm{(N)}}$. The determination of the model parameters was based on the new knowledge of the effects induced by acid deposition on ecosystems and the subsequent biogeochemical processes in China [14,31–33] (see Method in the Supplementary Data). Similarly, the CL of acidification for hundreds of headwater streams across China was updated using a modified Steady-State Water Chemistry model (see Method in the Supplementary Data).

### Critical load exceedance

The atmospheric deposition of S and N onto China was modeled using the CMAQ/2D-VBS air quality simulation system [24,34] (see Method in the Supplementary  Data).

The CL exceedances were calculated by subtracting the CL from the corresponding acid deposition. The exceedances of eutrophication CL were calculated as:


(6) \begin{eqnarray*}
{\mathrm{Ex}}\left( {{\mathrm{C}}{{\mathrm{L}}}_{{\mathrm{eut}}}} \right){\mathrm{ =\, }}{{\mathrm{N}}}_{{\mathrm{dep}}} - {\mathrm{\ C}}{{\mathrm{L}}}_{{\mathrm{eut}}}\left( {\mathrm{N}} \right)
\end{eqnarray*}

The exceedances of acidity CL were calculated as:


(7) \begin{eqnarray*}
{\mathrm{Ex}}\left( {{\mathrm{C}}{{\mathrm{L}}}_{{\mathrm{aci}}}} \right) &=& \left( {{{\mathrm{N}}}_{{\mathrm{dep}}} - {\mathrm{C}}{{\mathrm{L}}}_{{\mathrm{min}}}\left( {\mathrm{N}} \right)} \right){\mathrm{ \times }}\left( {{\mathrm{1}} - {f}_{{\mathrm{de}}}} \right) \\
&&+\, {{\mathrm{S}}}_{{\mathrm{dep}}} - {\mathrm{C}}{{\mathrm{L}}}_{{\mathrm{max}}}\left( {\mathrm{S}} \right)
\end{eqnarray*}

where the negative value indicates no exceedance in Equations (6) and (7).

### Uncertainty and sensitivity analysis

The uncertainties of CL and CL exceedances were mainly attributed to the errors or uncertainties of modeled input parameters when the uncertainties regarding the model structure and CL concept were not discussed. According to previous studies in China, the CLs are highly dependent on the Bc deposition [35] and soil weathering rates [36]. Here the uncertainty of Bc deposition was assumed to be 40%, according to the Normalized Mean Bias between the Bc modeling and monitoring. The uncertainty of Bc_w_ was assumed to be normally distributed with an SE of 20%, according to previous uncertainty analysis [35]. For further calculation of CL exceedances, the uncertainty comes from acid deposition modeling. The uncertainties for wet deposition of S and N were about –30% and –35%, respectively. Based on the error propagation, the uncertainties of CL exceedances were ∼40%. However, it is difficult to determine the uncertainty of the proportion of area with CL exceedances. Given the important impacts of Bc deposition and climate on CL computing [35], we investigate the sensitivity of CL to decreasing Bc deposition and climate changes (see Supplementary Data).

## Supplementary Material

nwae007_Supplemental_File

## Data Availability

The data that support the findings of this study are available from the corresponding authors upon reasonable request.
