# Supplementary material for: A probe into the acid deposition mitigation path in China over the last four decades and beyond
Source: Natl Sci Rev. 2024 Jan 5;11(4):nwae007. doi: 10.1093/nsr/nwae007 (PMC10941815; doi:10.1093/nsr/nwae007)
Supplement: nwae007_Supplemental_File [file nwae007_supplemental_file.docx]

Supplementary information for

**A probe into acid deposition mitigation path in China over the last four decades and beyond**

**Qian Yu^#1, 2^, Xiaodong Ge^#1^, Haotian Zheng^1^, Jia Xing^1, 3^, Lei Duan^1, 3*^, Dongwei Lv^1^, Dian Ding^1^, Zhaoxin Dong^1^, Yisheng Sun^1^, Posch Maximilian^3^, Danni Xie^4^, Yu Zhao^5^, Bin Zhao^1,2^, Shuxiao Wang^1,2^, Jan Mulder^6^, Thorjørn Larssen^7^, Jiming Hao^1,2*^**

^1^State Key Laboratory of Environmental Simulation and Pollution Control, School of Environment, Tsinghua University, Beijing 100084, China.

^2^State Key Laboratory of Pollution Control & Resource Reuse and School of the Environment, Nanjing University, Nanjing 210023, China.

^3^State Environmental Protection Key Laboratory of Sources and Control of Air Pollution Complex, Tsinghua University, Beijing 100084, China.

^4^International Institute for Applied System Analysis (IIASA), Laxenburg A-2361, Austria.

^5^School of Land Engineering, Chang’an University, Shaanxi 710064, China

^6^Faculty of Environmental Sciences and Natural Resource Management, Norwegian University of Life Sciences, Ås Box 5003, NO-1432, Norway.

^7^Norwegian Institute for Water Research, Oslo 0349, Norway.

^#^Contributing equally

*Corresponding Author. e-mail: [lduan@tsinghua.edu.cn](mailto:lduan@tsinghua.edu.cn) (LD); hjm-den@tsinghua.edu.cn

**This document includes**

Supplementary Methods

Table S1

Figures S1-S7

# **Supplementary Methods**

## Detail data sources

Trends of S and N deposition (Figure S1) were summarized from the published data and measurements across China.[1] For instance, the total S deposition in 1980-2004 was 1.6 times of the wet deposition, which was the product of annual rainfall and volume-weighed concentrations of SO_4_^2-^ in precipitation in 74 sites of China Meteorological Administration (CMA; 1992-2020; http://s.cma.gov.cn/zfxxgk/gknr/qxbg/), in 5 sites of Integrated Monitoring Program on Acidification of Chinese Terrestrial Systems (IMPACTS; 2001-2004)[2], in 7 sites of Acid Deposition Monitoring Network in East Asia (EANET; 2001–2018; https://www.eanet.asia/), and the review of previous studies [1, 3]. In 2005-2020, the total S deposition was derived from the combinate calculation of emissions inventory and air quality measurements and statistical modelling[4]. The total NO_3_^-^-N and NH_4_^+^-N deposition has been summarized in previous studies [5, 6]. Specifically, we used the national average value from Yu et al. (2019)[5] and from Wen et al. (2020)[6]. for N depositions in the periods of 1980-2015 and 2016-2018, respectively. The values of precipitation pH in China came from the values of 74 sites of China Meteorological Administration (CMA; 1992-2020; http://s.cma.gov.cn/zfxxgk/gknr/qxbg/).

For comparing with the development of acid deposition in Europe and North America, we collected the data for GDP, energy consumption, acidity gas emissions, S, N, and base cations (Bc) deposition, as well as precipitation pH since 1970 in EU-28 and the USA (Figures 1 & S1; Table S1). The GDP and energy consumption were derived from the International Energy Agency (IEA, https://www.iea.org/data-and-statistics). The data for emissions of SO_2_, NO_X_, and NH_3_ in 1990-2020 in the USA and EU-28 were the public data from the U.S. Environmental Protection Agency (EPA, https://www.epa.gov/air-emissions-inventories/air-pollutant-emissions-trends-data) and the European Environment Agency (EEA, https://www.eea.europa.eu/themes/air), respectively. For 1970-1990, the emissions of SO_2_, NO_X_, and NH_3_ were derived from the Emissions Database for Global Atmospheric Research (EDGAR, <https://edgar.jrc.ec.europa.eu/>) The precipitation pH and Bc deposition (only wet deposition) in the USA were the average values of the annual data from the sites of the National Trends Network (NTN) of the National Atmospheric Deposition Program (NADP, <https://nadp.slh.wisc.edu/networks/national-trends-network/>). And we got the total deposition of S, NO_3_^-^-N, and NH_4_^+^-N in the USA for 1989-2017 from the Clean Air Status and Trends Network (CASTNET, <https://www.epa.gov/castnet>). As no total deposition data were reported in CASTNET from 2018 to 2020, we calculated the total depositions using the wet deposition data in NADP and assumed the dry depositions were the same as those in 2017. The total deposition data for Europe were derived from the European Monitoring and Evaluation Program, EMEP, (<https://projects.nilu.no/ccc/index.html>), which is a scientifically-based and policy-driven program under the Convention on Long-range Transboundary Air Pollution (CLRTAP) for international co-operation to solve transboundary air pollution problems (<https://unece.org/environment-policy/air>). Furthermore, the critical loads for soil acidification, surface water acidification, and eutrophication and their exceedances in Europe were derived from the EMEP reports in 2021[7] and the Coordination Centre for Effects final report in 2017 [8], the International Cooperative Program report on Assessment and Monitoring Effects of Air Pollution on Rivers and Lakes [9], and Towards Cleaner Air. Scientific Assessment Report 2016 [10].

**Figure S1｜Trends of economy and atmospheric deposition in China, Europe, and the USA in 1980-2020.** The Bc deposition in China only included Ca^2+^ and Mg^2+^ from both natural and anthropogenic sources. However, the natural Bc depositions was set as fixed values with no interannual variation due to the limited basic data. Thus, the variations of Bc depositions in this study were derived from the changes of anthropogenic source.

**Table S1｜The current status and changes of economy and energy, acidifying pollutants emissions, acid depositions,** **and critical load exceedance (CL ex) in Europe, USA, and China.** The numbers indicate the current values in 2020, and the bars show the changes in the current values compared to those in 1990 in Europe and America and compared to those in 2005 in China. Blue and orange bars indicate decrease and increase trends, respectively.

^a^The changes need to multiply by 10;

^b^SW means surface water.

## Update of critical loads for soils in China

We used Simple Mass Balance (SMB) model to update the national CL for soil acidification and eutrophication with 1×1 km^2^ resolution. The model parameters update and the changes of critical loads for soils were described in detail below:

**Model parameters update.** The update of critical loads for soils was conducted based on improved model parameters, e.g., soil weathering rate, Gibbsite equilibrium constant, denitrification rate, nitrogen fixation rate, and the plant uptake rate of base cation and nitrogen. A new 1×1 km^2^ spatial distribution map of soil types derived from the Resource and Environment Science and Data Center (<https://www.resdc.cn/data.aspx?DATAID=145>) was used in the update of critical loads for soils. The standard of soil classification refers to the Second Soil Survey of China, which distinguishes 61 soil types. Similarly, the new Chinese Vegetation Map with a total of 796 vegetation subgroups distinguished at a scale of 1:1 M was acquired and resampled to 1 km^2^ using the nearest neighbor method [13]. Furthermore, the Chinese Runoff Map at a scale of 1:4 M was acquired and converted to a 1-km^2^ raster image[14]. The Gibbsite equilibrium constant *K*_gibb_ is now considered as a function of soil organic matter content[8]. And a new 1 km map of soil organic matter content derived from the national Tibetan Plateau Data Center was used in this study [15].

For base cation weathering, mineralogy data of major soil types were used to calculate the base cation weathering rate with the PROFILE model [16]. Considering that CaCO_3_ might be depleted during the long-term acid deposition, it was excluded from the calculation unless the content was higher than 0.5% according to the CaCO_3_ content data from the Food and Agriculture Organization of the United Nations (FAO) [17]. The weathering rate of all soil types was calculated with the same values of parameters except mineralogy ones. Then the results of the PROFILE model were corrected for soil moisture, surface area, and temperature using the following equation [8]:

$\text{Bc}_{\text{w}}\text{ = }\text{C}_{\text{sw}}\text{ ×}\frac{\text{SSA}}{\text{1.1×1.2}}\text{×}\exp\text{(}\frac{\text{A}}{\text{T}}\text{-}\frac{\text{A}}{\text{281}}\text{)}\text{×}\text{Bc}_{\text{w0}}$ (S1)

where C_sw_ is the correction factor for soil moisture, which ranges from 0.7 to 1.3 according to the soil moisture content from low to high. SSA is the soil-specific surface area (10^6^ m^2^/m^3^) estimated by the following equation [18]:

$\text{SSA=}\left( \text{0.3}\text{X}_{\text{sand}}\text{+2.2}\text{X}_{\text{silt}}\text{+8.0}\text{f}_{\text{clay}}\text{X}_{\text{clay}} \right)\text{×}\left( \text{1-S} \right)\text{×}\text{ρ}_{\text{soil}}$ (S2)

$\text{f}_{\text{clay}}\text{=1-}\frac{\text{X}_{\text{clay}}^{\text{2.5}}}{\text{X}_{\text{clay}}^{\text{2.5}}\text{+0.35}}$ (S3)

where X_sand_, X_silt,_ and, X_clay_ stand for the sand, silt, and clay content in the fine earth respectively and S stands for the fraction of the coarse soil. $\rho_{\mathrm{soil}}$ is the soil bulk density (10^3^ kg m^-3^). 1.1 and 1.2 in Eq. (S3) means 1.1×10^6^ m^2^/m^3^ and 1.2×10^3^ kg/m^3^，which are the values using in the PROFILE model. *A* in Eq. (S3) is 3600 K and *T* is the soil temperature (K). Data on soil basic properties was from FAO (2019)[18]. Soil temperature data were acquired from the National Tibetan Plateau Data Center [19]. It should be noted that the temperature here should not be the average annual temperature but the monthly average because it is an exponential relationship. In other words, we used the monthly temperature correction and then take the average, otherwise, the result would be underestimated.

For soil N transformation processes, nitrogen immobilization was estimated as the soil total nitrogen divided by soil age. Soil total nitrogen data were obtained from the National Tibetan Plateau Data Center [20]. The soils were divided into Skeletol Primitive Soils, Ferralisols, and others according to their average ages. Their ages were set to 1500 years, 130000 years, and 5000 years respectively [21]. Moreover, observed data on denitrification for main forest types in China can be accessed [22, 23]. We calculated the denitrification fraction *f*_de_ based on the observed data. The *f*_de_ in the other areas was determined according to the soil drainage status, ranging from 0 for excessively drained soils to 0.8 for very poorly drained soils. Soil drainage data was taken from FAO [18]. The SSA was also used to correct the denitrification fraction to prevent overestimating the fraction in some areas with coarse soils (setting *f*_de_ = 0.1 when SSA < 2×10^6^ m^2^/m^3^). The results are in good agreement with the observed data.

For net growth uptake, vegetation uptake was estimated from the net primary productivity (NPP) and the element content. The element content for major vegetation types refers to Duan et al.[24]. The NPP data simulated by GLO-PEM was taken from Resource Environmental Science and Data Center[25].

**The** **changes of critical loads for soils.** We derived the national scale critical load maps for soil in China many years ago, based on investigations on weathering rates of soils, uptake rates of vegetation, and depositions of base cations [24, 26, 27]. That critical load maps had a low spatial resolution (i.e., 1°× 1°) and large uncertainty due to the rough basic data. For instance, the soil texture was determined according to the soil type without consideration of the difference in soil moisture [24]. In consideration of improved model parameters, e.g., soil weathering rate, the Gibbsite equilibrium constant, denitrification rate, nitrogen fixation rate, and the plant uptake rate of base cation and nitrogen, we updated the national maps of critical loads for S and N with 1×1 km resolution (Figures S2 a & b). Lower critical loads for S were found in the forested northeast (< 0.2 keq ha^-1^ yr^-1^) with low temperature and thus low weathering rate, and the in the ferrallitic soils of Yunnan-Guizhou Plateau (< 0.2 keq ha^-1^ yr^-1^) and south (0.2-0.5 keq ha^-1^ yr^-1^) with both low weathering rate of soil (due to low content of weatherable minerals) and high vegetation uptake of Bc. The CL for S in the farmland-covered northern and unvegetated northwestern China were generally higher than 2.0 keq ha^-1^ yr^-1^ resulting in a high weathering rate and natural Bc deposition. On contrary, the critical loads for N were lower in the north, northwestern, and Tibet Plateau with poor vegetation uptake of N, while higher in the south with a considerable denitrification rate. In addition to the improvement of resolution, the precision has been enhanced compared to the original critical load maps of Duan et al. (2000) [24]. For instance, considering the impacts of temperature and humidity on the weathering rate, the underestimate of critical loads for S was revised in northeast China, and the critical loads for S also changed to relatively higher values in the north and northwest due to the correction of calcium carbonate content of soil (Figure S2c). With the amendment of the primary productivity of vegetation, we revised the overestimate of critical loads for N in the northwest, northern, and Tibet Plateau, and the underestimate of critical loads for N in south China (Figure S2d). Generally, China’s ecosystem was more sensitive to the N deposition than S deposition, manifesting as larger areas with lower critical loads for N, e.g., the region with critical loads for N lower than 0.4 keq ha^-1^ yr^-1^ accounting for 30% of the national area. The potential risk of eutrophication from N deposition seems more serious than acidification.

**Figure S2｜Updated critical load maps for soil in 2015. a**. critical load for S (CLmax(S)). **b.** critical load for N (minimum of CLmax(N) and CLeut(N)). **c**. comparison of the updated critical load for S with the previous version. **d**. comparison of the updated critical load for N with the previous version. The previous critical load was established by Duan et al. (2000)[24].

## Calculation of critical loads for surface waters

The critical loads of surface water across China were estimated based on a large-scale survey of 350 headwater streams from 2010 to 2020. The streams were distributed in the alpine meadow in northwest China, the temperate broadleaf forest and temperate coniferous forest in north and northeast China, as well as subtropical broadleaf and coniferous forests in the southwest, central south, and southeast China. Almost all the streams had pH values larger than 6.0 [28, 29]. The critical load has been calculated using the steady-state water chemistry (SSWC) model based on the establishment of Bc concentration before industrialization mainly from soil weathering in China [28]. The Bc deposition from anthropogenic sources was taken into account, due to commonly high Bc deposition in China, with a considerable fraction of anthropogenic origins, such as cement production, coal combustion, and intensive construction activities [26, 30, 31]. And the assumption of a linear relationship between Bc deposition and S and N deposition was used to estimate the Bc deposition, because of the limited information on the Bc emission and deposition. Here, we updated the critical loads for these streams using the renewed Bc deposition [32], and they are shown in Figure S3.

During the calculation of critical load for surface waters, the reduction of sulfate and denitrification were taken as sinks for S and N, especially in the subtropical zone [22, 23, 29, 31, 33, 34]. According to our S cycling study in the subtropical forests [35], the fraction of reduction to total S deposition was assumed as 0.3 in subtropical China. Field monitoring showed that the denitrification fraction of forest soils ranges from 0.20 (temperate coniferous forest) to 0.35 (subtropical broadleaf forest) in China [22, 23]. According to the critical load mapping in Europe [8], the denitrification ratios were set as 0.1 for the ecosystem without monitoring data, e.g. the alpine meadow in Northwest China.

**Figure S3｜** **Distributions of critical loads for 350 headwater streams across China**. The grey background shows soil pH in China (derived from the World Harmonious Soil Database by the Food and Agriculture Organization of the United Nations, FAO, and the International Institute for Applied Systems Analysis (IIASA).

## Deposition modeling

The atmospheric deposition of S and N onto China was modeled using the CMAQ/2D-VBS air quality simulation system [36, 37]. The anthropogenic air pollutant emission inventory used in the simulation was compiled at Tsinghua University [38-41]. Model parameters were verified by satellite and ground observation data [39]. The uncertainty of the wet deposition simulation was described by the coefficient of variation (the ratio of the standard deviation of the probability distribution to the average value). The wet deposition of sulfur and nitrogen were about -30% and -35%, respectively. Other model features, such as vertical resolution, physical and chemical schemes, model boundary conditions and initial conditions, and the mapping of anthropogenic emissions to the model grid, etc. were adopted from Zhao et al. (2018)[39]. The grid resolution of the deposition simulation domain was 36 km × 36 km, and the simulation results were interpolated to a resolution of 0.1°× 0.1° to obtain the N and S deposition map of China. The N and S deposition value at the stream water sampling points was obtained by the value of its grid (ArcMap 10.2, ESRI Company, Redlands, California, USA). The depositions in 2005, 2010, 2015, and 2022 were simulated in this study. Furthermore, two future scenarios for 2035, a combination of energy policies to meet the Nationally Determined Contribution pledged in the Paris Agreement and end-of-pipe controls under current legislation (2035-NDC), and a combination of co-benefit energy policies and end-of-pipe controls with maximum feasible reduction (2035-CBE), were set to model the depositions in the future. The national emissions of SO_2_, NO_X_, and NH_3_ will decrease by 62%, 51%, and 18%, respectively, compared with in 2015, under the 2035-NDC scenario, and decreased by 78%, 70%, and 18%, respectively, under the 2035-CBE scenario [36] (Figure S4a). The changes of depositions were showed in Figure S4 b & c.

**Figure S4｜** **Changes of emissions and depositions in China. a**, Emissions relative to 2005. **b**, Percentage of decrease in depositions in 2035 CBE compared to 2005. **c**, Changes of depositions during periods of 2005-2015 and 2015-2035 CBE.

## Impacts of Bc deposition decrease on critical loads

In addition to the critical load’s update in 2015, considering that the Bc deposition changes during the 2005-2015 periods and in the future scenarios, we calculated the critical load maps under in 2005, 2010, 2035-NDC, and 2035-CBE with the changeable Bc deposition. It showed that the decline of Bc deposition had significant impacts on a critical load of S, especially in East China, where most abatement occurs (Figure S5a). By contrast, the critical load of N was less affected, due to it depending on nutrient N rather than acidifying N (Figure S5b).

**Figure S5｜Changes of critical loads under the 2035-CBE scenario due to the reductions of Bc deposition. a**, for S**. b,** for N.

## Impacts of climate change on critical loads

Critical loads are steady-state quantities but can be influenced significantly by changes in temperature and precipitation induced by climate change. The effect of climate change on the geographical pattern and magnitude of critical loads and exceedances should be considered with great caution. Multiple interactions between climate change and biodiversity have been explored in general [42, 43]. Important interactions between the effects of air pollution and climate change should perhaps include considerations of the (potential) change of land cover [44], changes of localized ecosystems [45], or the combination of different environmental drivers (including N deposition) on the trade-off between global biodiversity and local diversity [46]. Firstly, temperature affects the weathering rate of base cation, which in turn affects the critical loads:

$\text{Bc}_{\text{w2}}\text{ =}\text{ Bc}_{\text{w1}}\text{×}\exp\left( \frac{\text{A}}{\text{T}_{\text{1}}}-\frac{\text{A}}{\text{T}_{\text{2}}} \right)$ (S4)

where A is 3600 K, and$\text{ Bc}_{\text{w1}}$ and $\text{Bc}_{\text{w2}}$ are the weathering rate in T_1_ and T_2_, respectively. Weathering rate also depends on soil moisture, which would change as the climate:

${\text{B}\text{c}}_{\text{w2}}\text{ = }\text{C}_{\text{sw}}\text{× B}\text{c}_{\text{w1}}\text{ }$ (S5)

where C_sw_ is a correction coefficient determined by the change in soil moisture. Secondly, runoff flux (Q) depends on precipitation and evapotranspiration, which is also determined by climate parameters. Q influences the critical ANC leaching (Eq. (2)), partially compensating for the influence caused by temperature change. Besides, in areas where the net uptake (${\text{B}\text{c}}_{\text{u}}$) is higher than the input base cation (${\text{B}\text{c}}_{\text{dep}}\text{+}\text{ Bc}_{\text{w}}$), we set:

$\text{Bc}_{\text{u}}\text{= }{\text{max\{B}\text{c}}_{\text{dep}}\text{+}\text{ Bc}_{\text{w}}-\text{Q ×}\text{ C}_{\text{limit}}\}$ (S6)

where C_limit_ is the minimum concentration of base cation in the runoff flux (0.01 eq/m^3^), below which they cannot be taken up by vegetation. Thirdly, base cation and nitrogen uptake are affected by changes in NPP induced by climate change:

$\text{M}_{\text{U}\text{u}\text{2}}\text{ }\text{=}{\text{ }\text{M}}_{\text{U}\text{u}\text{1}}\text{×}\frac{\text{NPP}_{\text{2}}}{\text{NPP}_{\text{1}}}$ (S7)

where M stands for nitrogen or base cations.

According to the Intergovernmental Panel on Climate Change (IPCC), compared to 1850-1900, the global surface temperature increased by 0.99 °C in 2001-2020 and is likely to be higher by 1.0 °C to 5.7°C in 2081-2100 [47]. However, climate-induced changes are generally not explicitly considered in the previous calculation of critical loads to evaluate the recovery of ecosystems from elevated acidic deposition in China. We used the “Representative Concentration Pathways” (RCPs) from IPCC Fifth Assessment Synthesis Report[48] to project the future climate change in the 21_st_ century. The RCPs included a stringent mitigation scenario (RCP2.6), two intermediate scenarios (RCP4.5 and RCP6.0), and one scenario with very high greenhouse gas emissions (RCP8.5). Among these, RCP4.5 and RCP6.0 are both steady-state conforming to the critical load concept, while RCP4.5 have stronger changes in the energy mix, which is consistent with the future energy policies in consideration of the“Carbon neutrality and peak” policy in China. Therefore, the RCP4.5 scenario was selected to represent the scenario that aims to keep global warming likely between 1.1°C to 2.6°C at the end of the 21_st_ century (2081-2100), compared to 1850-1900.

The critical loads beyond RCP4.5 scenario [48] were calculated in consideration of the impacts of climate change on the NPP, soil temperature, and moisture, as well as the runoff (Figure. S6). The model parameters of the future scenarios except deposition were developed by Beijing Climate Center based on the Coupled Model Intercomparison Project Phase 5 (CMIP5). We calculated the change (rates) of these parameters and interpolated them to a resolution of 1 km × 1 km to fit our basic data. Then we computed the values of these parameters under climate change with the change rates. This method solved the problem of inconsistency between the basic data used in the model and this study.

**Figure S6｜Increase of parameters induced by climate change. a**, soil temperature. **b,** soil moisture **c**, NPP. **d**. runoff.

**Figure S7｜Reduction of NH_3_ emissions under the 2035-EA scenario compared to the 2035-CBE scenario**.

# **Supplementary Reference**

1. Yu Q, Duan L. Chapter 8 Contribution of Atmospheric Reactive Nitrogen to Acid Deposition in China. In: Liu X, Du E (eds.). *Atmospheric Reactive Nitrogen in China: Emission, Deposition and Environmental Impacts*: Springer; 2020.

2. Larssen T, Duan L, Mulder J. Deposition and leaching of sulfur, nitrogen and calcium in four forested catchments in China: implications for acidification. *Environmental Science & Technology*. 2011; **45**(4): 1192-1198. doi: 10.1021/es103426p

3. Yu H, He N, Wang Q *et al.* Development of atmospheric acid deposition in China from the 1990s to the 2010s. *Environmental Pollution*. 2017; **231**: 182-190. doi: 10.1016/j.envpol.2017.08.014

4. Zhao Y, Xi MX, Zhang Q *et al.* Decline in bulk deposition of air pollutants in China lags behind reductions in emissions. *Nature Geoscience*. 2022; **15**(3): 190-+. doi: 10.1038/s41561-022-00899-1

5. Yu G, Jia Y, He N *et al.* Stabilization of atmospheric nitrogen deposition in China over the past decade. *Nature Geoscience*. 2019; **12**(6): 424-+. doi: 10.1038/s41561-019-0352-4

6. Wen Z, Xu W, Li Q *et al.* Changes of nitrogen deposition in China from 1980 to 2018. *Environment International*. 2020; **144**. doi: 10.1016/j.envint.2020.106022

7. Hilde F, Svetlana T, David S *et al.* *Transboundary particulate matter, photo-oxidants, acidifying and eutrophying components.* Norwegian Meteorological Institute; 2021. (EMEP Report 1/2021) (DHHS publication no.: Report Number)| (GPO o. Document Number)|.

8. Hettelingh JP, M.; Slootweg, J. *European critical loads: database, biodiversity and ecosystems at risk*. Bilthoven, Netherlands: Coordination Centre for Effects; 2017. (CCE Final Report 2017

) (DHHS publication no.: Report Number)| (GPO o. Document Number)|.

9. Austnes K, Aherne J, Arle J *et al.* *Regional assessment of the current extent of acidification of surface waters in Europe and North America*. Oslo: Norwegian Institute for Water Research; 2018. (ICP Waters Report 135/2018) (DHHS publication no.: Report Number)| (GPO o. Document Number)|.

10. Maas RG, P. . *Towards Cleaner Air. Scientific Assessment Report 2016*. Oslo: EMEP Steering Body and Working Group on Effects of the Convention on Long-Range Transboundary Air Pollution; 2016 (DHHS publication no.: Report Number)| (GPO o. Document Number)|.

11. Rafaj P, Amann M, Siri J *et al.* Changes in European greenhouse gas and air pollutant emissions 1960-2010: decomposition of determining factors. *Climatic Change*. 2014; **124**(3): 477-504. doi: 10.1007/s10584-013-0826-0

12. Adalibieke W, Zhan X, Cui X *et al.* Decoupling between ammonia emission and crop production in China due to policy interventions. *Global Change Biology*. 2021; **27**(22): 5877-5888. doi: 10.1111/gcb.15847

13. Crawford JT, Hinckley E-LS, Neff JC. Long-Term Trends in Acid Precipitation and Watershed Elemental Export From an Alpine Catchment of the Colorado Rocky Mountains, USA. *Journal of Geophysical Research-Biogeosciences*. 2020; **125**(11). doi: 10.1029/2020jg005683

14. Xiong Y, Zhang, J.Z. *Hydrological Regionalization in China.* Beijing, China: Science Press, 1995.

15. Dai Y, Shangguan, W. Dataset of soil properties for land surface modeling over China. In: Center TPD (ed.)2019.

16. Duan L, Hao JM, Xie SD *et al.* Determining weathering rates of soils in China. *Geoderma*. 2002; **110**(3-4): 205-225. doi: 10.1016/s0016-7061(02)00231-8

17. Panagos P, Van Liedekerke M. Harmonized World Soil Database v 1.0. *JRC Data Catalogue*. 2016.

18. Phelan J, Belyazid S, Kurz D *et al.* Estimation of Soil Base Cation Weathering Rates with the PROFILE Model to Determine Critical Loads of Acidity for Forested Ecosystems in Pennsylvania, USA: Pilot Application of a Potential National Methodology. *Water Air and Soil Pollution*. 2014; **225**(9). doi: 10.1007/s11270-014-2109-4

19. Meng X, Wang H, Lei X *et al.* Hydrological modeling in the manas river basin using soil and water assessment tool driven by CMAD. *Tehnicki Vjesnik-Technical Gazette*. 2017; **24**(2): 525-534. doi: 10.17559/tv-20170108133334

20. Shangguan W, Dai Y, Liu B *et al.* A China data set of soil properties for land surface modeling. *Journal of Advances in Modeling Earth Systems*. 2013; **5**(2): 212-224. doi: 10.1002/jame.20026

21. Kukla G. Saalian supercycle, Mindel/Riss interglacial and Milankovitch's dating. *Quaternary Science Reviews*. 2005; **24**(14-15): 1573-1583. doi: 10.1016/j.quascirev.2004.08.023

22. Fang Y, Koba K, Makabe A *et al.* Microbial denitrification dominates nitrate losses from forest ecosystems. *Proceedings of the National Academy of Sciences*. 2015; **112**(5): 1470-1474.

23. Yu L, Mulder J, Zhu J *et al.* Denitrification as a major regional nitrogen sink in subtropical forest catchments: Evidence from multi-site dual nitrate isotopes. *Global Change Biology*. 2019; **25**(5): 1765-1778. doi: 10.1111/gcb.14596

24. Duan L, Xie SD, Zhou ZP *et al.* Critical loads of acid deposition on soil in China. *Water Air and Soil Pollution*. 2000; **118**(1-2): 35-51. doi: 10.1023/a:1005197414074

25. Skjelkvale BL, Stoddard JL, Jeffries DS *et al.* Regional scale evidence for improvements in surface water chemistry 1990-2001. *Environmental Pollution*. 2005; **137**(1): 165-176. doi: 10.1016/j.envpol.2004.12.023

26. Zhao Y, Duan L, Larssen T *et al.* Simultaneous assessment of deposition effects of base cations, sulfur, and nitrogen using an extended critical load function for acidification. *Environmental Science & Technology*. 2007; **41**(6): 1815-1820. doi: 10.1021/es060380+

27. Zhao Y, Duan L, Xing J *et al.* Soil Acidification in China: Is Controlling SO2 Emissions Enough? *Environmental Science & Technology*. 2009; **43**(21): 8021-8026. doi: 10.1021/es901430n

28. Lv D, Yu Q, Xie D *et al.* Critical loads of headwater streams in China using SSWC model modified by comprehensive F-factor. *Science of the Total Environment*. 2022; **802**. doi: 10.1016/j.scitotenv.2021.149780

29. Yu Q, Zhang T, Cheng Z *et al.* Is surface water acidification a serious regional issue in China? *Sci Total Environ*. 2017; **584**: 783-790. doi: 10.1016/j.scitotenv.2017.01.116

30. Larssen T, Carmichael GR. Acid rain and acidification in China: the importance of base cation deposition. *Environ Pollut*. 2000; **110**(1): 89-102. doi: 10.1016/s0269-7491(99)00279-1

31. Duan L, Yu Q, Zhang Q *et al.* Acid deposition in Asia: Emissions, deposition, and ecosystem effects. *Atmospheric Environment*. 2016.

32. Zhao W, Zhao Y, Ma M *et al.* Long-term variability in base cation, sulfur and nitrogen deposition and critical load exceedance of terrestrial ecosystems in China. *Environmental Pollution*. 2021; **289**. doi: 10.1016/j.envpol.2021.117974

33. Yu L, Zhu J, Mulder J *et al.* Multiyear dual nitrate isotope signatures suggest that N‐saturated subtropical forested catchments can act as robust N sinks. *Global Change Biology*. 2016.

34. Yu Q, Zhang T, Ma X *et al.* Monitoring Effect of SO2 Emission Abatement on Recovery of Acidified Soil and Streamwater in Southwest China. *Environ Sci Technol*. 2017; **51**(17): 9498-9506. doi: 10.1021/acs.est.7b01147

35. Yu Q. Fate of deposited sulfur and nitrogen in typical subtropical forest ecosystems Southern China *Doctor*. Tsinhhua University, 2019.

36. Xing J, Lu X, Wang S *et al.* The quest for improved air quality may push China to continue its CO2 reduction beyond the Paris Commitment. *Proceedings of the National Academy of Sciences of the United States of America*. 2020; **117**(47): 29535-29542. doi: 10.1073/pnas.2013297117

37. Zhao B, Wang S, Donahue NM *et al.* Quantifying the effect of organic aerosol aging and intermediate-volatility emissions on regional-scale aerosol pollution in China. *Scientific Reports*. 2016; **6**. doi: 10.1038/srep28815

38. Wang SX, Zhao B, Cai SY *et al.* Emission trends and mitigation options for air pollutants in East Asia. *Atmos Chem Phys*. 2014; **14**(13): 6571-6603. doi: 10.5194/acp-14-6571-2014

39. Zhao B, Zheng H, Wang S *et al.* Change in household fuels dominates the decrease in PM2.5 exposure and premature mortality in China in 2005-2015. *Proceedings of the National Academy of Sciences of the United States of America*. 2018; **115**(49): 12401-12406. doi: 10.1073/pnas.1812955115

40. Zheng B, Tong D, Li M *et al.* Trends in China's anthropogenic emissions since 2010 as the consequence of clean air actions. *Atmos Chem Phys*. 2018; **18**(19): 14095-14111. doi: 10.5194/acp-18-14095-2018

41. Zheng B, Zhang Q, Geng G *et al.* Changes in China's anthropogenic emissions and air quality during the COVID-19 pandemic in 2020. *Earth System Science Data*. 2021; **13**(6): 2895-2907. doi: 10.5194/essd-13-2895-2021

42. Franklin J, Serra-Diaz JM, Syphard AD *et al.* Global change and terrestrial plant community dynamics. *Proceedings of the National Academy of Sciences of the United States of America*. 2016; **113**(14): 3725-3734. doi: 10.1073/pnas.1519911113

43. Posch M. Impacts of climate change on critical loads and their exceedances in Europe. *Environmental Science & Policy*. 2002; **5**(4): 10.

44. Pitelka LF, Gardner RH, Ash J *et al.* Plant migration and climate change. *American Scientist*. 1997; **85**(5): 464-473.

45. Barnosky AD, Hadly EA, Bascompte J *et al.* Approaching a state shift in Earth's biosphere. *Nature*. 2012; **486**(7401): 52-58. doi: 10.1038/nature11018

46. Bernhardt-Roemermann M, Baeten L, Craven D *et al.* Drivers of temporal changes in temperate forest plant diversity vary across spatial scales. *Global Change Biology*. 2015; **21**(10): 3726-3737. doi: 10.1111/gcb.12993

47. Change IPoC. *Climate Change 2021 The Physical Science Basis*. 2021. (IPCC AR6 Reports) (DHHS publication no.: Report Number)| (GPO o. Document Number)|.

48. Change IPoC. *Climate Change 2014*. 2014. (IPCC AR5 Synthesis Report) (DHHS publication no.: Report Number)| (GPO o. Document Number)|.
